# Editorial: Multiple Sclerosis and Neuroimmunology—Case Report Collection, Volume I

**DOI:** 10.3389/fneur.2022.976913

**Published:** 2022-07-13

**Authors:** Robert Weissert

**Affiliations:** Department of Neurology, University of Regensburg, Regensburg, Germany

**Keywords:** autoimmunity, central nervous system, multiple sclerosis, autoimmune encephalitis, COVID-19, SARS-CoV-2, HSV2, peripheral nervous system

## Introduction

Why does Frontiers in Multiple Sclerosis and Neuroimmunology publish Case Reports? There are several reasons. First, we can learn from case reports regarding novel disease entities and rare diseases. Second, case reports can give insight into better pathophysiological understanding of rare diseases. Third, case reports can be of interest regarding translational aspects and modeling in animal models. Fourth, case reports can be important regarding exploration of novel treatment regimen. Possibly, more reasons in favor of case reports can be found, the above indicated are most relevant for the Speciality Chief Editors of Frontiers in Multiple Sclerosis and Neuroimmunology. Of course, it is important that the presented cases bring additional value to the field. The ideal type of case report would contain data regarding the clinical manifestation, immunological, genetic, and pathological aspects of the disease and a novel treatment approach would be presented. Also, the findings should be discussed in the context of the relevant literature. Ideally, there should be two or more similar patients per case report. This is not trivial, especially if the case reports are coming from an individual center. Sometimes, case reports are the entrance into research for the involved physicians especially early in career, since the case has initiated a scientific argument. Therefore, the value of case reports also in gaining scientific thinking should not be underestimated. Frontiers of Multiple Sclerosis and Neuroimmunology is proud to present the “Multiple Sclerosis and Neuroimmunology—Case Report Collection I”. This collection contains 27 case studies that were published between 2021 and 2022. We would like to thank the Associate and Review Editors who have evaluated the manuscripts and significantly contributed with their constructive criticism.

## Topics of the 27 published case reports of “multiple sclerosis and neuroimmunology – case report collection I”

The 27 case reports of “Multiple Sclerosis and Neuroimmunology—Case Report Collection I” can be ordered as follows: (1) Reports regarding neurological manifestations of coronavirus disease 2019 (COVID-19). (2) Case reports regarding vaccination against severe acute respiratory syndrome coronavirus 2 (SARS-COV-2) and nervous-system related side effects. (3) Case reports with novel aspects of the emerging field of autoimmune encephalitis (AE). (4) Case reports of viral encephalitis. (5) Case reports regarding autoimmune peripheral nervous system disease. (6) Reports of cases of neuroimmunological side effects by pharmacological treatment. (7) Case reports regarding rare disease variants leading to demyelination within the central nervous system (CNS). (8) Case reports of rare disease variants successfully treated by immune therapy.

## Reports regarding neurological manifestations of COVID-19 and long-COVID

There are several neurological manifestations that can be associated with COVID-19 and post- and long COVID. Ishaq et al. present a case of a patient with opsoclonus myoclonus syndrome a rare neurological disease entity that was successfully treated with intravenous immunoglobulins (i.v. Ig). Gilio et al. present findings regarding the overlap of functional neurological disorders with long COVID. They indicate that stress and inflammation might drive disease precipitation of functional neurological disorders. Kimura et al. reports a patient with Bickerstaff brainstem encephalitis possibly triggered by COVID-19 and emerging Takotsubo cardiomyopathy (TC). They indicate that TC should be considered early when hemodynamic status remains unstable in patients with Bickerstaff brainstem encephalitis.

## Case reports regarding vaccination against SARS-COV-2 and nervous-system related side effects

Maniscalco et al. present a patient with multiple sclerosis (MS) with a relapse shortly after vaccination with BNT162b2 from Pfizer-BioNTech. Nistri et al. present 16 cases regarding relapse manifestation of MS triggered by vaccination against SARS-COV-2 with 10 patients vaccinated with BNT162b2 from Pfizer-BioNTech, two patients vaccinated with mRNA-1273 from Moderna and four patients vaccinated with ChAdOx1 from AstraZeneca. In a case series by Ancau et al. three patients are reported with acute hemorrhagic encephalomyelitis (AHE) after SARS-COV-2 vaccination with ChAdOx1 from AstraZeneca. The authors indicated that these vaccination-related neurological diseases are rare events. They argue for the need of a robust post-vaccination surveillance.

## Case reports with novel aspects of the emerging field of autoimmune encephalitis

Different types of autoimmune encephalitis (AE) are rare diseases with varying clinical presentations. Much can be learned regarding neurobiology from these diseases. Song et al. report a patient with coexistence of anti-alpha-amino-3-hydroxy-5-methyl-4-isoxazolepropionic acid (AMPA) receptor encephalitis and biomarkers of Alzheimer‘s disease. There are AE that are antibody-negative. Possibly, the relevant antigen has not been defined for these disease entities so far. Park and Kim present a patient of antibody negative AE that was successfully treated with B cell depletion by rituximab arguing for an underlying immunological disease-driving pathophysiology. Beside autoimmunity as driver for AE, there is paraneoplasia as disease initiator of AE, mediated by the anti-neoplasia-directed immune response. Gogia et al. report a case with amphiphysin antibody-associated stiff limp syndrome and myelopathy in a patient with breast cancer. Fang, Pan, et al. report a patient with relapses of anti-AMPA encephalitis with progressive brain atrophy and speculate regarding the underlying mechanisms. They observe partial functional recovery even in presence of severe brain atrophy after treatment with immunotherapy. Vaux et al. present a patient that was diagnosed with schizophrenia and was subsequently identified as a patient with anti-N-methyl-D-aspartate (NMDA) receptor encephalitis. Treatment with immune therapy led to improvement of symptoms in this patient. This case argues that patients with psychiatric diagnosis should be routinely explored for AE as a potential cause of their disease. This is important since immunotherapy can lead to improvement of symptoms and can in some patients even result in cure. Hashimoto‘s encephalopathy is a highly debated and questioned disease entity. Amano et al. present a patient with Hashimoto‘s encephalopathy associated with lymphomatosis cerebri and periodic synchronous discharges resembling prion, namely Creutzfeld-Jacob disease.

## Case report of virus-induced encephalitis

Kolesnik et al. report a patient with herpes simplex virus 2 (HSV-2) encephalitis presenting with the clinical manifestation of chorea. This is a very rare clinical manifestation in HSV-2 encephalitis.

## Case reports regarding autoimmune peripheral nervous system disease

Huang et al. report a patient with Guillain-Barré syndrome (GBS) and unilateral facial palsy. They summarize the current literature regarding such clinical presentations and report 28 cases. They speculate regarding the underlying immune response. Belgrado et al. had two patients with GBS and posterior reversible encephalopathy (PRES). They speculate that autoimmune dysregulation associated with GBS may be a trigger factor for co-emergence of PRES. A patient with reported combined central and peripheral demyelination (CCPD) was presented by Alshamrani et al. This patient had the coexistence of radiologically isolated syndrome (RIS) and Miller-Fisher-syndrome (MFS). Most so far reported cases of CCPD were diagnosed as having co-existence of MS and chronic inflammatory demyelinating polyradiculoneuropathy (CIDP).

## Reports of neuroimmunological side effects by pharmacological treatment

Koska et al. (a) report a patient with MS with severe lymphopenia due to treatment with fingolimod. The discontinuation of fingolimod treatment led to clinical deterioration with presence of neuropsychological symptoms that was difficult to overcome. Progressive multifocal leukoencephalopathy (PML) should be also taken early into consideration regarding differential diagnosis in such patients. Tang et al. present a patient with recurrent encephalopathy after treatment with ornidazole. Ornidazole is an antibiotic used for the treatment of protozoan infections. The patient recovered after discontinuation of the treatment with ornidazole. Longitudinal extensive transverse myelitis (LEMS) evolved and novel autoantibodies emerged in a patient treated with pemprolizumab reported by Charabi et al. The humanized antibody pemprolizumab targets programmed cell death protein 1 (PD1) on lymphocytes. Pemprolizumab is used in the treatment of various types of cancers. The emergence of LEMS was B cell mediated. A putative novel autoantigen was hypothesized in this disease condition that has not been defined so far.

## Case reports regarding rare disease variants leading to demyelination within the CNS

The expression of a proliferation inducing ligand (APRIL), belonging to the TNF superfamily, in the CNS was investigated in a patient with neuromyelitis optica (NMO) reported by Baert et al. APRIL provides a favorable environment for plasmocytes in the NMO lesion. In addition, APRIL induces an anti-inflammatory response in the NMO lesion. This indicates that targeting of APRIL by novel immunological therapies should only be executed with extreme caution since there is a dichotomy of APRIL-related functions in the NMO lesion. Differential diagnosis can be challenging in patients with atypical clinical presentations. Fang, Tong, et al. report a patient with primary CNS lymphoma that was initially misdiagnosed with glial fibrillary acidic protein (GFAP) astrocytopathy. Early diagnosis to differentiate these diseases is important, due to grossly different treatment approaches. Gao et al. report a patient with GFAP astrocytopathy associated with an area postrema syndrome. Ma et al. report a patient with bilateral meningo-cortical involvement of anti-myelin-oligodendrocyte-glycoprotein (MOG) IgG associated disease. Štourač et al. present the difficulties in the diagnosis and treatment of progressive tumefactive demyelination. The presented patient had an unfavorable outcome. Neurosarcoidosis can be difficult to diagnose. Braun et al. present a patient with myelopathy that was finally diagnosed with neurosarcoidosis.

## Case reports of rare disease variants successfully treated by immune therapy

Koska et al. (b) report a patient with Marburg variant of MS who was successfully treated with cyclophosphamide and ocrelizumab. Eculizumab was successfully used to treat a patient with seronegative NMO by Digala et al. This argues for a role of complement in seronegative NMO.

## Conclusion

The case series “Multiple Sclerosis and Neuroimmunology—Case Report Collection I” demonstrates the power of case studies as well as their limitations. Possibly, the cases will help to gain progress regarding clinical, pathophysiological, immunological, and treatment-related understanding in clinical and research environments dealing with related patient groups and evolving scientific topics in neuroimmunology. Interested physicians, physician-scientist and researchers will possibly have a benefit that will help to transform scientific thinking in medicine to a patient-focused research approach regarding disease biology and rationally well-founded medical and rehabilitative treatments.

## Author contributions

RW outlined and wrote the editorial.

## Conflict of interest

The author declares that the research was conducted in the absence of any commercial or financial relationships that could be construed as a potential conflict of interest.

## Publisher's note

All claims expressed in this article are solely those of the authors and do not necessarily represent those of their affiliated organizations, or those of the publisher, the editors and the reviewers. Any product that may be evaluated in this article, or claim that may be made by its manufacturer, is not guaranteed or endorsed by the publisher.

